# Expected impact of MRI-related interreader variability on ProScreen prostate cancer screening trial: a pre-trial validation study

**DOI:** 10.1186/s40644-020-00351-w

**Published:** 2020-10-09

**Authors:** Ronja Hietikko, Tuomas P. Kilpeläinen, Anu Kenttämies, Johanna Ronkainen, Kirsty Ijäs, Kati Lind, Suvi Marjasuo, Juha Oksala, Outi Oksanen, Tuomas Saarinen, Ritja Savolainen, Kimmo Taari, Teuvo L. J. Tammela, Tuomas Mirtti, Kari Natunen, Anssi Auvinen, Antti Rannikko

**Affiliations:** 1 Department of Urology, University of Helsinki and Helsinki University Hospital, PL900, 00029 HUS, Helsinki, Finland; 2 Research Program in Systems Oncology, Faculty of Medicine, University of Helsinki, Helsinki, Finland; 3 HUS Diagnostic Center, HUS Medical Imaging Center / Radiology, University of Helsinki and Helsinki University Hospital, Helsinki, Finland; 4 Department of Radiology, Tampere University Hospital, Tampere, Finland; 5 Department of Urology, Tampere University Hospital, Tampere, Finland; 6 HUSLAB Laboratory Services, Department of Pathology, HUS Helsinki University Hospital, Helsinki, Finland; 7 Faculty of Social Sciences, Tampere University, Tampere, Finland

**Keywords:** Prostate cancer, Agreement, Magnetic resonance imaging, PI-RADS version 2, Screening

## Abstract

**Background:**

The aim of this study is to investigate the potential impact of prostate magnetic resonance imaging (MRI) -related interreader variability on a population-based randomized prostate cancer screening trial (ProScreen).

**Methods:**

From January 2014 to January 2018, 100 men aged 50–63 years with clinical suspicion of prostate cancer (PCa) in Helsinki University Hospital underwent MRI. Nine radiologists individually reviewed the pseudonymized MRI scans of all 100 men in two ProScreen trial centers. All 100 men were biopsied according to a histological composite variable comprising radical prostatectomy histology (*N* = 38) or biopsy result within 1 year from the imaging (*N* = 62). Fleiss’ kappa (κ) was used to estimate the combined agreement between all individual radiologists. Sample data were subsequently extrapolated to 1000-men subgroups of the ProScreen cohort.

**Results:**

Altogether 89% men of the 100-men sample were diagnosed with PCa within a median of 2.4 years of follow-up. Clinically significant PCa (csPCa) was identified in 76% men. For all PCa, mean sensitivity was 79% (SD ±10%, range 62–96%), and mean specificity 60% (SD ±22%, range 27–82%). For csPCa (Gleason Grade 2–5) MRI was equally sensitive (mean 82%, SD ±9%, range 67–97%) but less specific (mean 47%, SD ±20%, range 21–75%). Interreader agreement for any lesion was fair (κ 0.40) and for PI-RADS 4–5 lesions it was moderate (κ 0.60). Upon extrapolating these data, the average sensitivity and specificity to a screening positive subgroup of 1000 men from ProScreen with a 30% prevalence of csPCa, 639 would be biopsied. Of these, 244 men would be true positive, and 395 false positive. Moreover, 361 men would not be referred to biopsy and among these, 56 csPCas would be missed. The variation among the radiologists was broad as the least sensitive radiologist would have twice as many men biopsied and almost three times more men would undergo unnecessary biopsies. Although the most sensitive radiologist would miss only 2.6% of csPCa (false negatives), the least sensitive radiologist would miss every third.

**Conclusions:**

Interreader agreement was fair to moderate. The role of MRI in the ongoing ProScreen trial is crucial and has a substantial impact on the screening process.

## Background

Early detection of aggressive prostate cancer (PCa) remains challenging. Prostate-specific antigen (PSA) -based screening reduces cancer-specific mortality by approximately 20% by detecting aggressive cancers at an early stage when they can be successfully treated. However, such screening also leads to overdiagnosis of clinically insignificant cancers that are likely to be subsequently overtreated [1]. Therefore, organized PCa screening has not been implemented in Europe.

Traditionally, the standard procedure for men with a clinical suspicion of PCa, with elevated PSA or abnormal digital rectal examination, has been the systematic 10- or 12-core transrectal ultrasound (TRUS) -guided biopsy [2, 3]. The limitations of this approach are that some cases of clinically significant PCa (csPCa) are not detected. In contrast, many cases of clinically insignificant PCa (cisPCa) are overdiagnosed using this approach, and all men with a clinical suspicion of PCa are required to undergo invasive and harmful biopsy procedure [4, 5].

Multiparametric magnetic resonance imaging (mpMRI) of the prostate and targeted biopsies of only lesions identified is a promising diagnostic pathway. A recent study by Drost and colleagues has shown that MRI improves the detection ratio (DR) of csPCa by 12% and decreases the risk of cisPCa diagnosis by 30–40% compared to systematic biopsies in men with a suspected PCa [5]. Of men with a clinical suspicion of PCa, roughly one-third have negative MRI and can therefore avoid prostate biopsy [5]. Thus, the MRI pathway is an appealing tool for PCa screening, as it may be possible to maintain the substantial reduction in PCa mortality and yet avoid the unnecessary biopsies and overdiagnosis of cisPCa.

However, the usefulness of MRI in a population-based screening is highly dependent on the quality of the MRI process (imaging and reporting) per se. The ability of MRI to detect csPCa has improved over the past decade as the Prostate Imaging Reporting and Data System (PI-RADS) was introduced in 2012 [6] and has been updated twice since then [7]. Nevertheless, several uncertainties remain, as the interreader agreement on PI-RADS categories is moderate at best and the experience of an individual radiologist may have an effect on the specificity of reporting [8].

The aim of our study is to evaluate the potential impact of MRI -related interreader variability in an ongoing ProScreen PCa screening trial. We initiated a population-based prospective randomized screening trial (ProScreen) in 2018, which is still ongoing. In ProScreen men with a suspicion of csPCa in a biochemical screening test (PSA and the four kallikrein test (4 K) (free PSA, intact and total PSA and kallikrein-like peptidase 2 [hK2]) are referred to mpMRI with targeted biopsies of the visual lesion(s) only [9].

The precision of the ProScreen trial at detecting csPCa while avoiding overdiagnosis, ultimately depends on the subjective evaluation of the MRI by the radiologist. In this current study, we specifically investigated the potential impact of prostate MRI-related interreader variability on the ProScreen trial.

## Methods

The ProScreen trial is a population-based screening trial with a total of 67,000 men aged 50–63 years who reside in Helsinki or Tampere in Finland that commenced in 2018 [9]. These men are randomized to either a screening arm or a control arm in a 1:3 ratio. The men in the screening arm are invited to consent for the trial and upon giving their informed consent, a serum PSA test is taken, whereas the men in the control arm will not be contacted. A PSA ≥ 3.0 μg/l value is considered abnormal and will trigger the next stage of screening, i.e. 4 K score test [10]. Men with a 4 K score ≥ 7.5% are referred for prostate MRI in one of the participating urology departments. Men with lesions that have a PI-RADS of 3–5 upon MRI are then invited for transrectal ultrasound guided fusion biopsies (FBx) of the target lesions only. Men with negative MRI are invited for TRUS guided systematic 12-core biopsies only when PSA density is ≥0.15 μg/l.

Here, we chose a retrospective sample of 100 non-consecutive men who had been referred to the Helsinki University Hospital (HUS) for suspicion of PCa before the initiation of the ProScreen trial to come up with different GGG classes of roughly equal size. Previous MRI or negative biopsies were allowed. Men had varying baseline risks for PCa. Most men (*n* = 91) had undergone MRI before diagnostic biopsies, whereas for nine men the MRI was used post-biopsy in cancer staging before definitive treatment. The 91 men were biopsied within 6 months of the MRI. The mean age at imaging was 67 years (SD ±9) and the median PSA level was 9.4 μg/l (interquartile range [IQR] 6.7–14.5 μg/l).

**Fig. 1:**
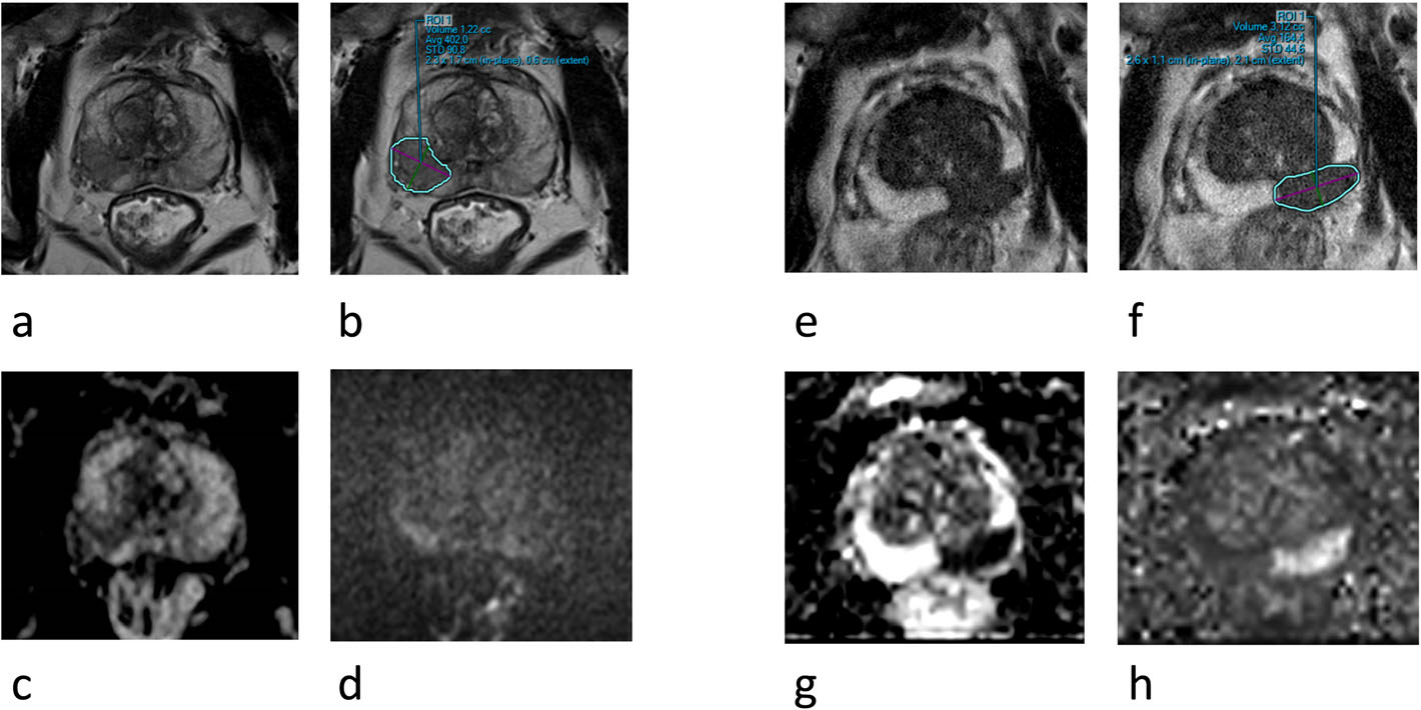
Prostate MRI images from two patients demonstrating poor agreement (images **a-d**) and good agreement (images **e-h).** T2 (**a** and **e**), T2 with delineated lesion (**b** and **f**), ADC (**c** and **g**) and high b (**d** and **h**) images are shown. In a 64-year old man with PSA of 22 ng/ml (**a-d**) five radiologist scored a lesion and four did not (fusion biopsies were benign and no PCa has been diagnosed during a 2.5-year follow-up). In a 72-year old man with PSA of 10.5 ng/ml all nine radiologist correctly scored a lesion (GG5 in fusion biopsies)

The imaging was performed with 3 T scanners Philips Achieva (from 2014) and with Siemens Skyra (from 2017). The protocol included T2 weighted imaging (T2WI), diffusion (DWI) with ADC-mapping and dynamic contrast enhancement (DCE). Surface coil was used and the slice thickness was 3 mm for T2WI and DWI, and 4 mm for DCE. The image resolutions for T2WI were 0,6 × 0,6 mm (Skyra) or 0,6 × 0,7 (Achieva). ADC-maps were calculated from diffusion b-values 0 (Achieva) or 50 (Skyra), 100 and 800. The high b-value images, b2000, for tumour detection were scanned separately (Achieva) or extrapolated up to b1600 by using lower b-value data (Skyra). DCE imaging comprised intravenous administration of gadolinium-based contrast agent (Dotarem®, 0,2 ml/kg, 2 ml/s) with the temporal resolution of 7 s (Skyra) or 8 s (Achieva) up to 2 min 30 s, and flip angle 12° (Skyra) or 10° (Achieva). The possible early enhancement was assessed visually, and the data were further processed by using scanner’s software (until 2015) or DynaCad to create signal intensity curves of suspected lesions (Fig. 1). Until October 2017 only summary reports of the DCE analyses were stored as jpg-images. These could not be pseudonymized adequately. Hence, for the re-evaluation in the present study, the original DCE data were not available. MRI images were first pseudonymized. Subsequently, the images of all 100 men were each made available to all nine radiologists that were concurrently reporting prostate MRI in the two ProScreen trial centers. Previous experience of the nine radiologists regarding prostate MRI reads varied from 40 to 100 reads (one radiologist) to 100–300 reads (one radiologist) to > 500 reads (seven radiologists) (see Table 1). The PSA concentration, the age of the patient and the 4 K score data were the only additional data the radiologists had during assessment. The radiologists were blind to all other relevant data regarding the patients of the sample. The MRI scans were evaluated using version 2 PI-RADS [11] but the DCE image sets were not available, thus the re-evaluation is based on only biparameric MRI (bpMRI).

**Table 1:**
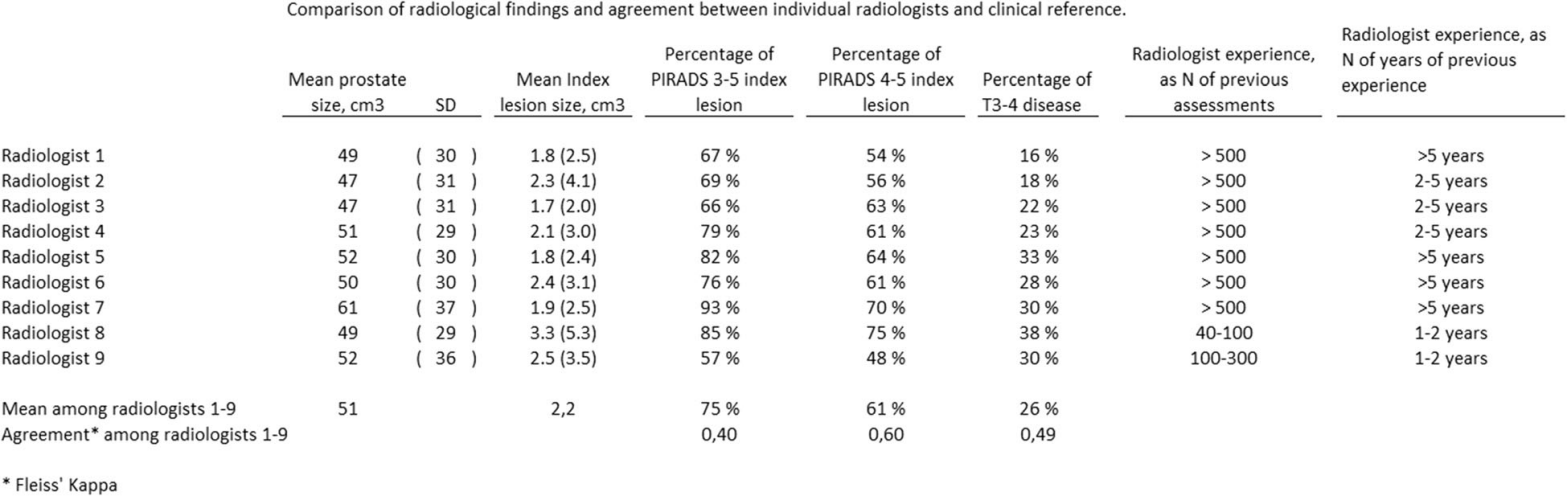
Comparison of radiological findings and agreement between individual radiologists and clinical reference

Structured pathological assessment was given using the five-tier Gleason Grade Groups (GGG): 3 + 3; 3 + 4; 4 + 3; 4 + 4 and > 8 [12]. We focused on the evaluation of the index lesion, which was defined as the largest and highest-grade lesion in the prostate [13]. The gold standard in sensitivity and specificity analyses was a histological composite variable: radical prostatectomy histology (for those who underwent radical prostatectomy, *n* = 38) or biopsy result within 1 year from the imaging (for those who did not undergo radical prostatectomy, *n* = 62). As the positive predictive value (PPV) and negative predictive value (NPV) are highly dependent on the underlying prevalence of the condition, we also report positive and negative likelihood (LH) ratios.

Fleiss’ kappa was used to estimate the combined agreement between all individual radiologists. Finally, the distribution of PI-RADS score was presented graphically for each patient stratified by composite pathological result. The study protocol was evaluated by the research ethics committee of the HUS Helsinki University Hospital (HUS/333/2019).

## Results

The median follow-up time for men with negative MRI or negative biopsies was 2.4 years (range 1.5–5.4 years and mean 2.6 years, SD ±0.82 years). Twenty-two men (22%) had both targeted and standard 12-core biopsies, 68 men (68%) had only targeted biopsies, nine (9%) men had only standard 12-core biopsies and one man had saturation biopsies. Most men (*n* = 87, 87%) had PCa upon biopsy. Of the 13 men (13%) with benign biopsy, two (15.4%) were later diagnosed with PCa. Of these two, one had GGG1 cancer diagnosed by transurethral resection of the prostate and the other one had GGG2 cancer diagnosed by subsequent saturation biopsies.

**Fig. 2:**
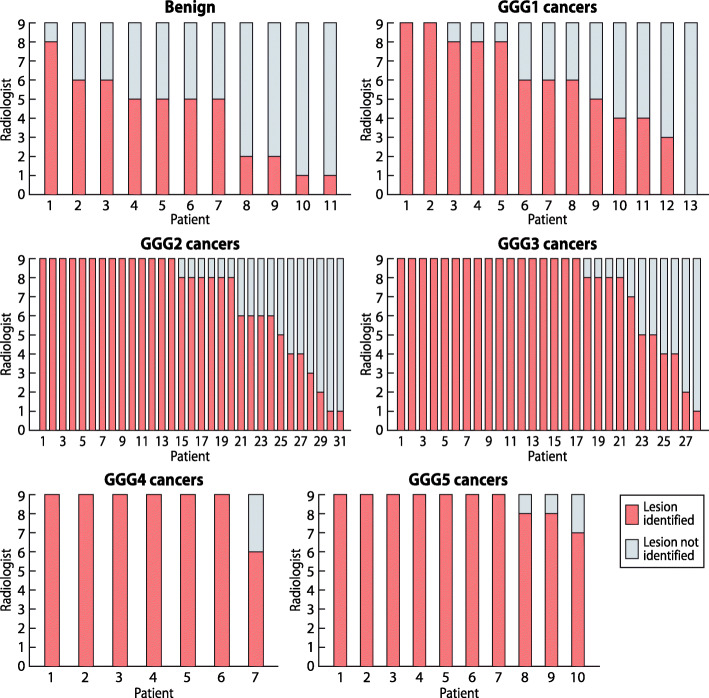
The number of radiologists identifying PIRADS 3–5 index lesion

Of the 89 men diagnosed with PCa, 13 (14.6%) had GGG1 cancer, 31 (34.8%) had GGG2 cancer, 28 (31.5%) had GGG3 cancer, 7 (7.9%) had GGG4 cancer and 10 (11.2%) had GGG5 cancer in the biopsies. Thus, 76% of the patients in the cohort sample had clinically significant cancer (GGG2 or worse) and roughly half had GGG3 cancer or worse. Patient level assessments grouped by pathological result are illustrated in Fig. 2.

### Agreement between radiologists

Individual radiologists reported a PI-RADS 3–5 index lesion in 75% of cases (range 57–93%) (Table 1). Agreement among radiologists was fair (Fleiss’ kappa 0.40). Agreement was moderate (Fleiss’ kappa 0.60) for PI-RADS 4–5 lesions, which were found in 61% of cases (range 48–75%) (Table 1). T3–4 disease was reported in 26% of cases (range 16–38%) and agreement among radiologists was moderate (Fleiss’ kappa 0.49) (Table 1).

### Radiological assessment compared to the pathological reference

The radiological assessment compared to the pathological reference is presented in Table 2. Mean positive and negative LR was 2.0 (SD ±1.1, range 1.1–4.0) and 0.4 (SD ±0.2, range 0.2–0.7), respectively.

**Table 2:**
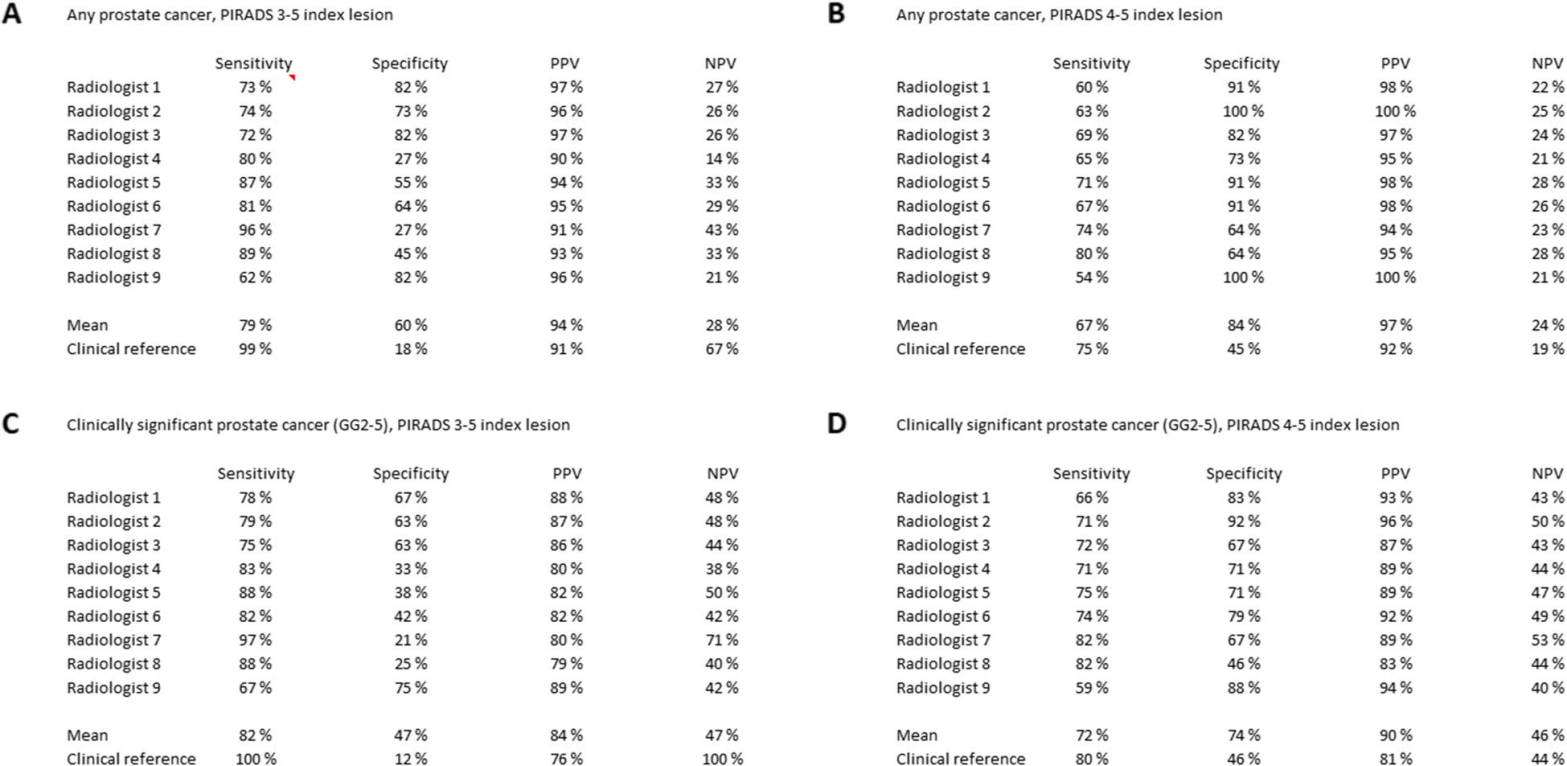
Sensitivity, specificity, positive predictive value and negative predictive value for PIRADS 3–5 or PIRADS 4–5 index lesions

For more suspicious lesions (PI-RADS 4–5 index lesion, any PCa) (Table 2b), corresponding positive and negative LR median was 4.2 (SD ±3.0, range 0–7.9) and 0.4 (SD ±0.1, range 0.3–0.5), respectively.

For clinically significant PCa (GGG2–5) and PI-RADS 3–5 index lesion (Table 2c), corresponding median and mean positive and negative LR was 1.5 (SD ±0.6, range 1.2–2.7 and 0.4 (SD ±0.1, range 0.1–0.5), respectively.

Positive and negative LR was 2.8 (SD ±2.1, range 1.5–8.9) and 0.4 (SD ±0.1, range 0.3–0.5), respectively, for PI-RADS 4–5 lesions in finding csPCa (Table 2d).

Seven of the nine radiologists were very experienced with prostate MRI assessment and therefore no comparative statistical analysis on experience level was done.

### Extrapolation to the screening cohort

In the ProScreen trial approximately 16,700 men are randomized to the screening arm. A power calculation determined that roughly 11,690 men would be expected to participate in screening (70%) and of these, 1520 men would have PSA ≥ 3.0 μg/l and subsequently 1000 men would have a 4KScore of ≥7.5% and would therefore have the indication for prostate MRI [9].

Assuming a 30% prevalence of csPCa in the screen-positive subcohort with 1000 men, the mean sensitivity (82%) and specificity (47%) of radiologists would entail 639 men being referred to biopsy of which 244 men would be true positive and 395 false positive. Of the 361 men who would not be referred to biopsy, 305 would be true negatives and 56 would harbor a cancer that would be missed, i.e., false negative.

If the sensitivity and specificity of the evaluations done by the most sensitive radiologist is assumed in a similar subcohort of 1000 men, 846 men would be biopsied (292 true positive; 554 false positive) and 154 men would not be biopsied (146 true negative; 8 false negative).

Conversely, if the sensitivity and specificity of the evaluations carried out by the least sensitive radiologist is extrapolated into the same subcohort of 1000 men, 376 men would be biopsied (of which 201 would be true positive; 175 false positive) and 624 men would not be biopsied (525 true negative; 99 false negative).

## Discussion

We estimated the impact of interreader variability on our ongoing ProScreen PCa screening trial which, in addition to the two objective biomarker measurements (PSA and 4 K), is ultimately dependent on subjective evaluation of the MRI by the radiologist. Even though our results correspond reasonably well to the published data [4, 5, 8], the range between the radiologists is broad. We observed a significant difference between radiologists in sensitivity and specificity which can have a substantial impact on the precision of the screening. Assuming a 30% prevalence for csPCa in screen positive men, twice as many men would be biopsied based on MRI interpretation by the most sensitive radiologist compared to the least sensitive radiologist and almost three times more men would undergo unnecessary biopsies (i.e. due to false positive screening results). Conversely, the most sensitive radiologist would miss only 2.6% of csPCa (false negatives), whereas the least sensitive radiologist would miss every third case. On average 64% of men would be biopsied and 62% of them would undergo unnecessary biopsy but every fifth man with csPCa would be missed.

We could not evaluate the explanatory factors for the observed variation between radiologists, although the radiologists’ experience of prostate MRI readings were collected. However, the majority (seven out of nine radiologists) were very experienced, which prevented us from comparing the impact of experience (Table 1). Interestingly, a recent study that evaluated MRI-related interreader variability reported that the sensitivity for detection of index lesion was not dependent on radiologist experience, whereas the specificity was highly dependent on reader experience [8]. Therefore, other causal factors of interreader variability might also exist. It can be assumed that the extremes (clearly malignant and clearly benign) would be reported more consistently although the area in-between these extremes would be more prone to interreader variability. This is at least in part supported by our data as most of the radiologists correctly identified GGG 4–5 cancers whereas there was substantial variability for men with cisPCa as none of the benign prostates were correctly identified by all the radiologists (Fig. 2). Such high variability leads to unnecessary biopsies and, thus, causes unnecessary morbidity and elevated costs.

We found fair interobserver agreement for the detection of index lesion (0.40) and moderate agreement (0.60) for the detection of PI-RADS 4–5 index lesion. While similar relatively modest agreement has been observed previously by Baldisserotto et al. (interobserver agreement of 0.53) [14] we were expecting better agreement [15, 16]. Greer et al. evaluated the interobserver agreement for five radiologist and found a high interobserver agreement [15]. Girometti et al. found substantial agreement in assessing PCa with category three or greater [16]. Greer et al. [8] reported excellent agreement (0.87) for detecting index lesion and substantial agreement (0.74) for true-positive findings. In that same study, nine radiologists evaluated on average 58 MRIs from a group of 163 patients of whom 110 (67%) had a subsequent radical prostatectomy as a reference standard. This might explain their better observed agreement as men selected for RP are more likely to harbor large csPCa. Furthermore, Greer and colleagues noted that not all radiologists interpreted all the images. Similar to the study by Greer et al., biopsy information was not available by the radiologists in our present study. In addition, the interpretation in our study was based on bpMRI as DCE was not available for the radiologists. It has been suggested that readers have a high level of agreement on DCE-MRI assessment in general [17] although agreement regarding the peripheral zone lesions on DCE images may be compromised [18]. It is anticipated that PI-RADS v2.1 will decrease the interreader variation in DCE MRI analyses and reduce overinterpretations compared to PI-RADS v2 [19]. It has also been reported that DCE may assist in the detection of csPCa in both the peripheral zone (PZ) and the transitional zone (TZ) [17, 20]. Therefore, we expect that the agreement in the ProScreen trial would be better as radiologists that interpret the MRIs of screen positive men in practice have the opportunity to consult colleagues on difficult cases and may have some benefit to evaluating DCE in men with equivocal peripheral zone lesions according to PI-RADS. On the other hand, concern regarding gadolinium deposition in the brain and the extra scanner time (i.e. costs) needed for DCE may promote the use of bpMRI [21]. Also, unknown factors could influence mpMRI interpretation, other than reader experience, and that this needs to be investigated in further studies. Eventually, further standardization in the parameters used for imaging may improve consistency of reporting.

Some evident discrepancies with radiological and pathological assessment were seen in our study (see Fig. 1). A man with a large GG3 pT3a cancer in prostatectomy specimen was correctly identified in MRI by only one of nine radiologists. Conversely, a man with only benign inflammatory histology in biopsies was erroneously scored as cancer by all but one radiologist. These cases aptly demonstrate the inability of prostate MRI to detect some 7% of the csPCas correctly, whereas it is well known that inflammatory changes may confound a reading by appearing suspicious in prostate MRI and is a common cause for false positives [4, 22, 23].

Currently, substantial uncertainty remains about the appropriate actions to be taken on men with clinical suspicion for PCa but negative MRI (nMRI). The true false negative rate, i.e., the rate at which csPCas are missed by MRI, is difficult to assess. Clinically the question is whether systematic biopsies should accompany targeted biopsies. For proper analysis of false negative rate, prostates of all men with PCa suspicion would be removed for pathological evaluation irrespective of MRI/biopsy results, which of course is unethical. The PROMIS trial tackled this dilemma by taking intense 5 mm template mapping biopsies on all men. They showed a false negative rate of 12% for >GGG1 cancers and 7% for >GGG2 cancers [4]. Another way to look at this would be to rely on csPCa incidence during follow-up after negative MRI. Panebianco V et al. showed that csPCa diagnosis free survival (DFS) was 95% after 2 years follow-up [24]. Furthermore, a recent analysis from another cohort largely corroborate this finding by reporting a csPCa DFS of 99.6% after 3 years [25]. In terms of the ProScreen trial this is reassuring as screen positive men (PSA > 3 and 4 K > 7.5%) with a nMRI or negative targeted biopsy are rescreened after 2 years. Furthermore, men with nMRI with PSAD (PSA density) > 0.15 will undergo systematic biopsies as supported by the recent review and meta-analysis [26].

The use of pre-biopsy MRI as a triage test criterion for restricting biopsy to only men with suspicious lesions, could result in one in four men avoiding biopsy. This is in line with the data from PROMIS and PRECISION trials where nMRI rates of 27 and 28% were reported [4, 27]. A recent Cochrane review reported up to one-third of men with nMRI [5] whereas up to one in two has been reported in some expert centers [28].

We found a moderate sensitivity for the detection of any PCa (79%) and approximately the same sensitivity for csPCa (ISUP GG 2–5, 81%). Even though the MRI was quite accurate in detecting csPCa, the sensitivity for more suspicious lesions (PI-RADS 4–5) did not improve. Other studies have obtained better sensitivities (Cochrane review 91%, PROMIS 93%) [4, 5]. This is possibly due to differences in reference standards. The Cochrane review was based on template-guided biopsy and the PROMIS trial was based on 5 mm template mapping biopsy as opposed to the systematic biopsy for most men used in our study. The specificity of the MRI for csPCa in our study was in concordance with the Cochrane review [5].

A low NPV for cisPCa and a high PPV were found in contrast to other studies [4, 5, 29]. The NPV and PPV are highly dependent on the underlying prevalence of the disease and the observed discrepancy, which probably reflects the high prevalence of csPCa (76%) in our study cohort sample.

The threshold used to define positive MRI is equivocal [6]. The intermediate PI-RADS 3 lesions are particularly difficult to define [5]. If the threshold in our study was set at PI-RADS 4 and 5 lesions instead of PI-RADS 3, the proportion of men with nMRI would have increased from 25 to 39%, and the MRI would not have correctly identified 33% of csPCa. This is in concordance with the literature [4, 8]. In respect to the ProScreen trial, it might be an acceptable compromise to increase further the ratio between the benefit and the harm due to built-in “safety tailgate”, whereby men with nMRI would undergo systematic biopsy if PSAD > 0.15, and otherwise (PSAD < 0.15) would be invited for the next screening round in 2 years [9].

+Some inherent limitations to our study must be considered. The PCa prevalence in our study cohort (87%) is higher than in the general population (30%) [30, 31]. Furthermore, the prevalence of csPCa was also high (76%). The prevalence of csPCa in the Cochrane meta-analysis, the MRI-FIRST trial and the 4 M trial were 28, 38 and 30%, respectively [5, 28, 29] hence these discrepancies largely restrict any direct comparison of the results. However, our study was not designed to assess the diagnostic performance of MRI, thus the related limitations such as high prevalence of the disease and verification bias are not essential. Instead, the aim of our study was to evaluate the interreader agreement between radiologists and extrapolate these to the ProScreen cohort. The agreement between radiologists was better for the very high-risk GGG4 and GGG5 cancers as opposed to men with benign histology, which is reassuring in regard to mortality reduction in ProScreen. Nevertheless, the consequence is that more benign prostates would be scored suspicious and thus, the cost-efficiency of screening will be reduced by the taking of unnecessary biopsies.

Though the aim and design of the study were to evaluate interreader agreement, we should also pay attention to the urologist’s role in the diagnostic work up. Accuracy of the fusion biopsy to detect the lesion correctly identified by the radiologist could not be evaluated here.

Re-reading the MRI images did not entirely mimic the routine clinical scenario for several reasons. Although they are still of controversial importance, the DCE sequences were not available for the radiologists [11, 32]. Moreover, contrary to clinical routine, radiologists were not allowed to consult a colleague with challenging cases. These likely underestimate the interreader agreement observed. Finally, nearly all radiologists were relatively experienced, and therefore we had no opportunity to study the effect of experience on the agreement dimension. All these factors may limit the extent to which the results can be generalized, although they should not have a significant effect on the ProScreen trial per se.

## Conclusions

The interreader variability among radiologists whom interpret prostate MRI is significant. In respect to the ongoing ProScreen PCa screening trial, the effect on mortality reduction is expected to be modest. However, poor interobserver agreement especially for men with true benign histology may cause undue sampling of the prostate and thus drive inefficacy of screening.

## Data Availability

Yes.

## Abbreviations

MRI: Magnetic resonance imaging
PCa: Prostate cancer
PSA: Prostate-specific antigen
csPCa: Clinically significant PCa
cisPCa: Clinically insignificant PCa
TRUS: Transrectal ultrasound
mpMRI: Multiparametric magnetic resonance imaging
DR: Detection ratio
PI-RADS: The Prostate Imaging Reporting and Data System
4 K: The four kallikrein
FBx: Fusion biopsies
T2WI: T2-weighted imaging
DWI: Diffusion-weighted imaging
ADC: Apparent diffusion coefficient mapping
DCE: Dynamic contrast enhancement
bpMRI: Biparameric MRI
GGG: Gleason Grade Groups
PPV: Positive predictive value
NPV: Negative predictive value
LH: Likelihood
PZ: Peripheral zone
TZ: Transitional zone
DFS: Diagnosis free survival
nMRI: Negative MRI
PSAD: PSA density
